# Food profile of Yanomami indigenous children aged 6 to 59 months from the Brazilian Amazon, according to the degree of food processing: a cross-sectional study

**DOI:** 10.1017/S1368980022001306

**Published:** 2022-05-27

**Authors:** Aline Oliveira dos Santos Moraes, Elma Izze da Silva Magalhães, Jesem Douglas Yamall Orellana, Giovanna Gatica-Domínguez, Paulo Augusto Ribeiro Neves, Paulo Cesar Basta, Juliana dos Santos Vaz

**Affiliations:** 1 Postgraduate Program in Nutrition and Food, Federal University of Pelotas, Pelotas, RS, Brazil; 2 Postgraduate Program in Public Health, Federal University of Maranhão, São Luis, MA, Brazil; 3 Leônidas and Maria Deane Institute, Oswaldo Cruz Foundation, Manaus, AM, Brazil; 4 Postgraduate Program in Epidemiology, Federal University of Pelotas, Pelotas, RS, Brazil; 5 Samuel Pessoa Department of Endemics, National School of Public Health, Oswaldo Cruz Foundation, Rio de Janeiro, RJ, Brazil; 6 Faculty of Nutrition, Federal University of Pelotas, Rua Gomes Carneiro, nº 1, 2º Andar, Sala 227, Centro, Pelotas, RS 96010-610, Brazil

**Keywords:** Infant feeding, Food consumption, Indigenous population, Indigenous health

## Abstract

**Objective:**

The current study aimed to characterise the food profile of Yanomami indigenous children according to the degree of food processing and its associated factors.

**Design:**

This is a cross-sectional study with Yanomami indigenous children aged 6 to 59 months. Socio-demographic, maternal and infant data were collected through a standardised questionnaire. The food profile was obtained by using a list of thirty-four foods to verify the child’s consumption of these foods on the day preceding the interview. Foods were classified according to the degree of processing based on the NOVA system (in natura or minimally processed, processed culinary ingredients, processed and ultra-processed). In natura and minimally processed foods were subdivided into ‘regional’ and ‘urban’ foods. Poisson regression analysis was applied to estimate the associated factors according to the 90 % CI.

**Setting:**

Three villages (Auaris, Maturacá and Ariabú) in the Yanomami indigenous territory, in the Brazilian Amazon.

**Participants:**

In total, 251 Yanomami children aged 6 to 59 months were evaluated.

**Results:**

The prevalence of consumption of ‘regional’ and ‘urban’ in natura or minimally processed foods was 93 % and 56 %, respectively, and consumption of ultra-processed foods was 32 %. Ultra-processed food consumption was 11·6 times higher in children of Maturacá and 9·2 times higher in Ariabú when compared with the children of Auaris and 31 % lower in children who had mothers with shorter stature.

**Conclusion:**

Despite the high frequency of consumption of in natura and minimally processed foods, the consumption of ultra-processed foods was substantial and was associated with demographic and maternal factors in Yanomani indigenous children under 5 years of age.

The high prevalence of chronic undernutrition and nutritional deficiencies observed in indigenous populations in Brazil is a hallmark of the social and nutritional inequalities in the country^(1,2)^. The First Brazilian National Survey on Indigenous People’s Health and Nutrition (2008–2009) revealed that 26 % of children under 5 years of age were stunted and that 51 % had anemia^(2)^. Nevertheless, recent studies have shown that short stature in relation to age affects more than 80 % of Yanomami children, thus revealing severe vulnerability and a nutritional deficiency in this population^(3,4)^.

Such problems result not only from the difficulty of producing or acquiring food but also from the historical violation of basic rights, precarious socio-economic conditions and land conflicts^(5)^. Traditionally, the Yanomami are considered hunters and gatherers^(6,7)^; however, the regular presence of invaders (especially loggers and prospectors) on their traditional territory drastically affects the areas used for hunting, fishing and gathering and, consequently, the availability and variety of the native food sources^(8,9)^, thus causing scarcity of these items^(8,9)^. Even without previous authorisation, some villages have been obliged to live with non-indigenous people from different parts of Brazil, including representatives of the Brazilian State as well as other kinds of invaders in their traditional territories. Among other consequences, this forced interaction has created a local scenario of socio-environmental vulnerability and has exposed almost all families, especially children, to the consumption of industrialised products and ultra-processed foods of low nutritional value, which are high-energy-dense, low in fibre and micronutrients and rich in preservatives and industrial additives^(1,7,10)^. Currently, the foods that mainly contribute to the energy intake of the Yanomami are acquired in regional markets, especially rice, tubers, beans, manioc flour and fruits^(10)^.

Previous studies carried out with indigenous children from Latin America have provided data on nutritional status, though with limited information on dietary indicators^(11–14)^. There are a few studies that have been carried out with indigenous children in Brazil that have reported monotonous diets^(13,14)^ that are quantitatively below nutritional needs^(15,16)^ and which contain a substantial presence of ultra-processed foods, such as soft drinks made from powder, soda, candy, bread, cookies, artificial juice, artificial yogurt, canned foods and instant noodles^(14,16)^, thus indicating the importance of more profound studies on this topic.

Evidence of a nutritional transition in Brazilian non-indigenous children between 6 and 59 months of age has revealed a consumption at least once a week of cookies, soft drinks and snacks^(17)^. A systematic review that included thirty-one studies that evaluated the diet of Brazilian children under 7 years of age concluded that the diet of this population was characterised by a high consumption of fried foods, soft drinks, sweets and salt^(18)^. No previous research on the food profile has been conducted on Yanomami children who live in isolated areas in the rainforest where there is limited contact with non-indigenous society.

Since the central and transformative promise of the 2030 Sustainable Development Goals Agenda is to ‘leave no one behind’^(19)^ and, in order to fill an important gap in the literature and show the vulnerable situation in which Yanomamis live, we aimed to characterise the food profile of indigenous Yanomami children aged from 6 to 59 months, according to the degree of food processing and to investigate its association with socio-economic, demographic, maternal and anthropometric factors. Our hypothesis is that Yanomami indigenous children living in regions in close contact with non-indigenous society consume more ultra-processed foods when compared with those living without contact with non-indigenous society.

## Methods

### Study area and population

**Fig. 1:**
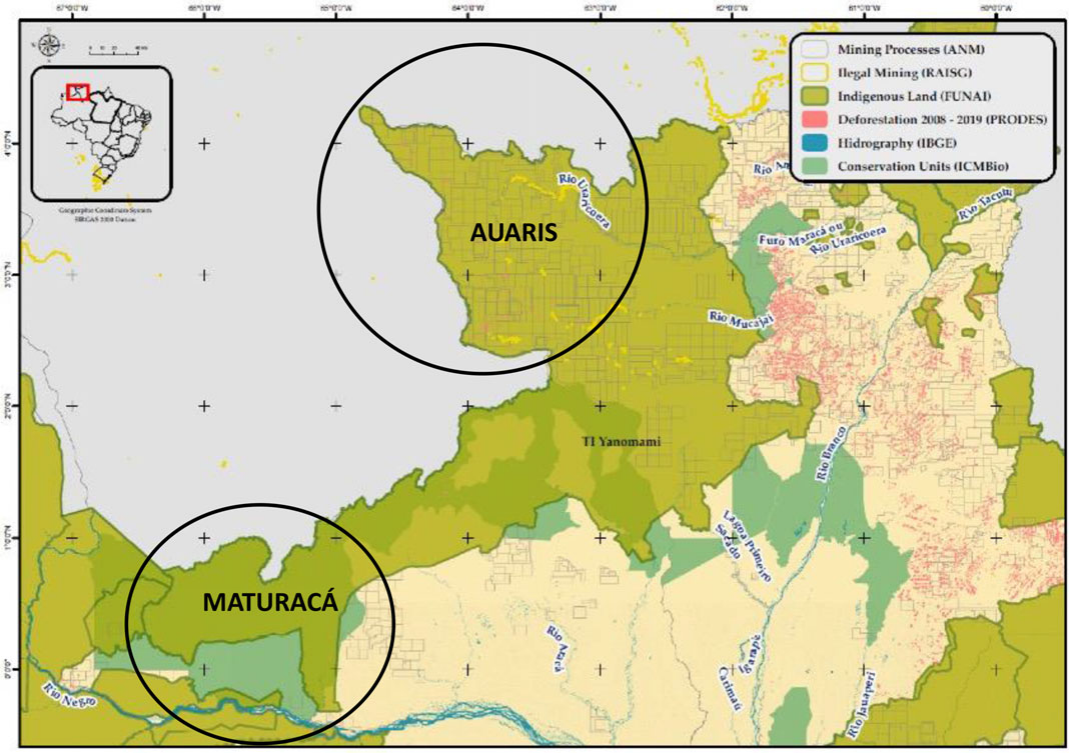
Map of the Yanomami indigenous territory, Brazilian Amazon

In the extreme north of Brazil, the Yanomami population is of approximately 27 000 individuals, who are distributed in more than 300 villages that are located in an area of 9 664 975 hectares^(20)^, which is known as the Yanomami Indigenous Territory. The current study was carried out in two administrative regions: (i) Auaris, which is located in the extreme north of the state of Roraima, with access exclusively by air from the state capital, Boa Vista and (ii) Maturacá, situated in the state of Amazonas, with access by air from Boa Vista, or by a combined land and boat trip from the municipality of São Gabriel da Cachoeira, Amazonas (Fig. 1). In the Auaris region, eight small villages (Koronau, Kolulu Guarape, Traira/Auaris Posto, Katimani, Amonokomaú, Grabi-I, Polibi and Laranjeira) were included. In the Maturacá region, two large villages (Ariabú and Maturacá) were included. For this reason, in the present study, three strata of comparison were used.

### Sample design and selection

This is a cross-sectional study based on a census of indigenous children under 5 years of age that was conducted between December 2018 and February 2019. Households with children under 5 years of age and their respective mothers/guardians were invited to participate in the current study. Initially, 304 children under 5 years of age comprised the sample. As complementary feeding is recommended at 6 months of age, we excluded fifty-three children under 6 months of age. The final sample consisted of 251 children aged 6 to 59 months: 39 % from Maturacá, 33 % from Ariabú and 28 % from Auaris.

### Data collection

Trained researchers conducted the interviews with mothers/guardians of children under 5 years of age during home visits and applied a structured questionnaire and took anthropometric measurements. When necessary, the interviews were simultaneously translated from Portuguese into the predominant language of the region, with the support of native interpreters.

### Food profile

The food profile was established by using a pre-structured questionnaire composed of a list of foods in order to verify the child’s consumption of these foods on the day preceding the interview. This questionnaire consisted of a list of thirty-four food items with objective questions (yes/no) for each of them, in addition to an item ‘others’ for the inclusion and description of foods not included in the list. The food list was elaborated after a formal planning meeting with anthropologists, nutritionists and health professionals who have long experience working in the target communities. This list was pre-tested with a group of Yanomami women and their children in Boa Vista, the capital of Roraima state, as well as with selected Yanomami leaders in the month preceding the field work. The thirty-four food items are presented in Table 1.

**Table 1:** List of the thirty-four food items that composed the food questionnaire applied to indigenous children aged 6 to 59 months. Yanomami indigenous territory, Brazil, 2018–2019

1	Banana	11	Tapir	21	Brazil nut	31	Milk powder
2	Manioc	12	Fish	22	Palm heart	32	UHT milk
3	Biju*	13	Crab	23	Manioc flour	33	Tinamou‡
4	Açaí†	14	Earthworm	24	Coconut	34	Curassow§
5	Yam/sweet potato	15	Sugarcane	25	Canned foods	Others (describe)	
6	Mushroom	16	Honey	26	Table salt		
7	Deer	17	Pumpkin	27	Sugar		
8	Monkey	18	Corn	28	Rice		
9	Snake	19	Peach palm	29	Vegetable oil		
10	Wild pig	20	Ant/larvae	30	Cookies		

* Biju is a starch flat cake made with manioc flour that is typical of indigenous of Amazon region.

† Brazilian fruit predominantly grown in the Amazon region, rounded, with a dark colour, ranging from purple to black, it grows in bunches and, predominantly, in places with more humid or flooded soils.

‡ Small-billed tinamou is a bird from the Amazon region, similar to the guinea fowl, brown in colour, it has a meat that is much appreciated, especially by the indigenous people.

§ A gallinaceous bird native to the forests of Central and South America, with a well-developed crest and black plumage with yellow (male) or brown or reddish tones (female).

Using the NOVA system^(21)^, the foods consumed were classified in the following manner according to their degree of processing: *in natura* or minimally processed foods (e.g. fruits, vegetables, meats and beans), processed culinary ingredients (e.g. vegetable oil, salt and sugar), processed foods (e.g. canned foods, processed meats and bread) and ultra-processed foods (food products designed to create low cost, long shelf-life, convenient and hyperpalatable products, e.g. chocolate powder, artificial juice, soft drink and noodles). *In natura* or minimally processed foods were subdivided into two groups: ‘regional’ (of indigenous preparation or food available naturally in the village/forest) or ‘urban’ (food from the city that is available in small food markets in the community and/or exchanges with non-indigenous people working in the villages). Honey and pepper are usually classified in the urban environment as processed culinary ingredients. Due to the way in which it is obtained and consumed among indigenous people, it was included in the group of *in natura* or minimally processed foods.

### Anthropometric assessment

Birth weight was obtained by consulting the birth certificate, child health booklets, vaccination cards or by consulting the demographic module of the Indigenous Health Care Information System. Children born with < 2500 g were classified as low birth weight^(22)^.

Maternal and infant anthropometric data (weight and length/height) were collected according to the techniques recommended by the Brazilian Ministry of Health^(23)^. To measure the weight in kilograms of children with the ability to stand and their mothers, a portable digital scale (Seca^®^, model 877) with a maximum capacity of 150 kg and precision of 0·1 kg was used. For infants, the mother–infant function was used, in which the baby was weighed on the mother’s lap and the mother’s weight was subtracted from the mother–infant weight.

The stature of children aged over 24 months and their mothers was measured using a vertical stadiometer (Alturexata^®^, 213 cm total extension, 1 mm precision). Children aged 24 months or less were measured in dorsal decubitus using an infantometer (Alturexata^®^ 108 cm total extension, 1 mm precision). Children were classified as stunted and adolescent mothers of short stature if they had *Z*-scores of length/height-for-age < 2 SD from the median reference population, according to the WHO^(24)^. For adult mothers (>19 years), the cut-off point for short stature was <145 cm^(25)^.

The nutritional status of the children was evaluated as height-for-age and BMI-for-age in *Z*-scores, calculated using WHO AnthroPlus^®^ software. The cut-off points established by the WHO^(24)^ were considered.

### Socio-economic, demographic, maternal and infant characteristics

The following socio-economic and demographic characteristics were considered and were categorised as follows: regular income (yes/no), conditional cash transfer program – *Bolsa Família* (yes/no), availability of a place for purchases of food in the community (yes/no), place of residence (Auaris/Maturacá/Ariabú) and number of residents in the household (≤9/≥10 people).

Regarding maternal characteristics, the following variables were considered and classified as follows: marital status (with partner; without partner), age (≤19/20–29/≥30 years) and short stature (yes/no).

The children’s characteristics and the variables of nutritional status were classified as follows: sex (male/female), age (6–23/24–59 months), birth weight (<2500/≥2500 g), stunted (no/yes) and BMI-for-age (thin/normal weight/risk of overweight/overweight/obesity).

### Statistical analyses

Absolute and relative frequencies were estimated according to socio-economic and demographic characteristics, maternal and infant characteristics, as well as food profile. Considering the particularities of feeding in childhood, the analyses were stratified by age group (6–23/24–59 months). The frequency of consumption of each food group (‘regional’ *in natura* or minimally processed, ‘urban’ *in natura* or minimally processed, processed and ultra-processed) was estimated according to socio-economic, demographic, maternal and child variables, with respective 90 % CI. Pearson’s *χ* ^2^ or Fisher’s exact tests were used to verify differences in the proportions of the food groups between the categories of each of the variables.

Due to changes in the dietary patterns of children in Brazil, as well as the negative impacts of ultra-processed food consumption on health^(26)^, the analyses were focused on the factors associated with the consumption of ultra-processed foods. *Poisson* regression analysis with robust variances was applied to estimate the crude and adjusted prevalence ratios with a 90 % CI. Variables with *P*-values <0·20 in the bivariate analysis were included in the multivariate analysis, considering a probability <10 % as a level of statistical significance in the final model. Since the study was based in hard-to-reach areas in the Brazilian Amazon with a small sample size, *P*-values of 0·10 were adopted to capture any potential association that due to small sample size would be disconsidered if a *P*-value of 0·05 was used. Furthermore, it is worth remembering that we carried out a census in the investigated villages and that all children under 5 years of age were included in the study. Data were analysed using Stata 14.0 software (StataCorp.).

## Results

Of the 251 children investigated, approximately 51 % were male and 73 % were between the ages of 24 and 59 months. Prevalence of low birth weight was 11 %. The overall prevalence of stunting was 91 %, of which 19 % were stunted and 72 % severely stunted. In general, the children had a normal BMI-for-age (77 %). Regarding maternal characteristics, most of the mothers (54 %) were between 20 and 29 years of age, had a short stature (73 %) and lived with a partner (91 %). Most households comprised up to nine residents (66 %) and were in the vicinity of small food markets (84 %). More than half of the families had no regular income (56 %) and did not participate in any government cash transfer program (59 %) (Table 2).

**Table 2:** Socio-economic, demographic, maternal and individual characteristics of indigenous children aged 6 to 59 months living in villages of the Yanomami indigenous territory, Brazil, 2018–2019 (*n* 251)

Variables	*n*	%
Current place of residence		
Auaris	70	27·9
Maturacá	98	39·0
Ariabú	83	33·1
Sex of the child		
Female	124	49·4
Male	127	50·6
Age of child		
6–23 months	67	26·7
24–59 months	184	73·3
Birth weight*		
< 2500 g	26	10·8
≥ 2500 g	215	89·2
Stunted		
No	23	9·2
Yes	228	90·4
BMI-for-age		
Thin	3	1·2
Normal weight	193	76·9
Risk of overweight	52	20·7
Overweight	2	0·8
Obesity	1	0·4
Maternal age†		
≤ 19 years	18	7·4
20–29 years	132	54·3
≥ 30 years	93	38·3
Maternal short stature‡,§		
No	63	26·7
Yes	173	73·3
Maternal marital status‖		
With partner	226	91·5
Without partner	21	8·5
Number of residents in the household¶		
≤ 9	163	66·3
≥ 10	83	33·7
Place for food purchases in the community**		
Yes	177	83·9
No	34	16·1
Residents with regular income††		
Yes	109	44·1
No	138	55·9
Cash transfer program (*Bolsa Família*)‡‡		
Yes	101	40·7
No	147	59·3

* 10 data missing.

† 8 data missing.

‡ 15 data missing.

§ Maternal short stature cut-off: *Z*-score ≤ -2 (women aged ≤ 19 years) and stature ≤ 145 cm (for women aged 19 years or more).

‖ 4 data missing.

¶ 5 data missing.

** 40 data missing.

†† 4 data missing.

‡‡ 3 data missing.

The items most reported in the group of *in natura* or minimally processed ‘regional’ foods were fruits (69 %), corn, roots or tubers (45 %); peach palm or palm heart (33 %); fish or crab (33 %) and biju or couscous (32 %). Among ‘urban’ foods, the most reported were black beans (29 %), rice or pasta (19 %), chicken (17 %), coffee or coffee with milk (15 %) and powdered cow’s milk (10 %). The most frequently reported ultra-processed foods were cakes or cookies (25 %) and chocolate or chocolate powder (6 %) (Table 3).

**Table 3:** Frequency of food consumption according to the degree of food processing of indigenous children aged 6 to 59 months living in villages of the Yanomami indigenous territory, Brazil, 2018–2019 (*n* 251)

Group/foods consumed	Total		6–23 months (*n* 67)		24–59 months (*n* 184)	
	*n*	%	*n*	%	*n*	%
*In natura* or minimally processed foods						
Regional	233	92·8	56	83·6	176	95·6
Fruits*	173	68·9	39	58·2	134	72·8
Corn, roots or tubers†	112	44·6	19	28·4	93	50·5
Peach palm or palm heart	83	33·1	16	23·9	67	36·4
Fish or crab	82	32·7	18	26·9	64	34·8
Biju	80	31·9	20	29·8	60	32·6
Wild meat‡	63	25·1	10	14·9	53	28·8
Chibé (manioc flour soup)	62	24·7	16	23·9	46	25·0
Sugarcane	43	17·1	11	16·4	32	17·4
Mushroom	26	10·4	5	7·5	21	11·4
Porridges (banana, manioc flour or corn)	23	9·2	11	16·4	12	6·5
Others§	66	26·3	28	41·8	38	20·6
Urban	141	56·2	38	56·7	103	56·0
Black beans	74	29·5	16	23·9	58	31·5
Rice or pasta	49	19·5	15	22·4	34	18·5
Chicken	42	16·7	9	13·4	33	17·9
Coffee or coffee with milk	38	15·1	7	10·4	31	16·8
Powdered cow’s milk	25	10·0	7	10·4	18	9·8
Others‖	14	5·6	6	8·9	8	4·3
Processed culinary ingredients	103	41·0	20	29·8	83	45·1
Sugar	87	34·7	15	22·4	72	39·1
Table salt	87	34·7	17	25·4	70	38·0
Vegetable oil	56	22·3	10	14·9	46	25·0
Processed foods	30	11·9	8	11·4	22	12·0
Canned foods or processed meats	25	10·0	7	10·4	18	9·8
Bread	7	2·8	–		7	3·8
Ultra-processed foods	80	31·9	19	28·4	61	33·1
Cakes or cookies	62	24·7	16	23·9	46	25·0
Chocolate or chocolate powder	15	6·0	2	3·0	13	7·1
Artificial juice or soft drink	9	3·6	1	1·5	8	4·3
Noodles	3	1·2	1	1·5	2	1·1

* Pineapple, açaí, banana, cocoa, coconut, cupuaçu, guava, ingá (*Inga edulis*), orange, watermelon, passion fruit and tucumã (*Astrocaryum aculeatum*).

† Corn, manioc and yam/sweet potato.

‡ Tapir, snake, agouti, monkey, curassow, small-billed tinamou, paca, wild pig, toad and deer.

§ Breast milk, vegetables and legumes, ant or larvae, honey, natural fruit juice, earthworm, pepper, vegetable/fish broth and Brazil nut.

‖ Beef or egg soup, oatmeal.

Consumption of ‘regional’ *in natura* or minimally processed foods and processed culinary ingredients was significantly higher among children ≥23 months. Consumption of ‘urban’ *in natura* or minimally processed, processed and ultra-processed foods was significantly higher in children from Maturacá and Ariabú (Table 4). Prevalence of ‘urban’ *in natura* or minimally processed foods, culinary ingredients, processed and ultra-processed foods was significantly higher in children of households with beneficiaries of the *Bolsa Família* cash transfer program and those who live in households near to small food markets. Prevalence of ‘urban’ *in natura* or minimally processed, processed and ultra-processed foods was significantly higher in children of mothers of adequate stature. Children with adequate birth weight had a higher prevalence of consumption of ‘urban’ *in natura* or minimally processed foods. Children of mothers without a partner had a significantly higher consumption of processed foods (Table 4).

**Table 4:** Prevalence and associations of food consumption according to the socioeconomic, demographic, maternal and individual characteristics of indigenous children aged 6 to 59 months living in villages of the Yanomami indigenous territory, Brazil, 2018–2019 (*n* 251)

Variables	*In natura* or minimally processed				Processed culinary ingredients		Processed		Ultra-processed	
	Regional		Urban		Regional		Urban		Urban	
	*P* value %	90 % CI	*P* value %	90 % CI	*P* value %	90 % CI	*P* value %	90 % CI	*P*-value %	90 % CI
Place of residence	0·32		< 0·001		< 0·001		0·01		< 0·001	
Auaris	95·7	89·3, 99·0	5·7	2·0, 12·6	14·3	8·0, 23·0	2·9	0·5, 8·7	7·1	2·9, 14·4
Maturacá	92·9	87·0, 96·6	81·6	74·0, 87·8	43·9	35·3, 52·7	15·3	9·7, 22·6	39·8	31·5, 48·6
Ariabú	89·2	81·8, 94·2	68·7	59·3, 77·0	60·2	50·6, 69·3	15·7	9·5, 23·7	43·4	34·1, 53·0
Sex of child	0·77		0·35		0·29		0·65		0·49	
Female	91·9	86·7, 95·6	53·2	45·4, 60·9	44·3	36·8, 52·1	12·9	8·3, 18·9	29·8	23·1, 37·3
Male	92·9	88·0, 96·2	59·1	51·4, 66·4	37·8	30·6, 45·4	11·0	6·8, 16·7	33·9	26·9, 41·4
Age of child	0·001		0·92		0·03		0·99		0·47	
6–23 months	83·6	74·3, 90·5	56·7	45·9, 67·0	29·8	20·7, 40·3	11·9	6·1, 20·5	28·4	19·4, 38·8
24–59 months	95·6	92·3, 97·8	56·0	49·6, 62·2	45·1	38·9, 51·4	12·0	8·2, 16·6	33·1	27·4, 39·3
Birth weight	0·70		0·10		0·99		0·99		0·02	
< 2500 g	96·1	83·0, 99·8	42·3	25·8, 60·2	42·3	25·8, 60·2	11·5	3·2, 27·2	11·5	3·2, 27·2
≥ 2500 g	91·6	87·8, 94·5	59·1	53·2, 64·7	42·3	36·7, 48·1	12·6	9·0, 16·9	33·5	28·2, 39·2
Maternal age	0·47		0·58		0·60		0·78		0·97	
≤ 19 years	88·9	69·0, 98·0	50·0	29·1, 70·9	44·4	24·4, 65·9	11·1	2·0, 31·0	33·3	15·6, 55·4
20–29 years	91·7	86·6, 95·3	56·1	48·5, 63·4	38·6	31·5, 46·1	10·6	6·5, 16·1	32·6	25·8, 39·9
≥ 30 years	94·6	89·0, 97·9	61·3	52·3, 69·8	45·2	36·3, 54·2	14·0	8·5, 21·3	31·2	23·3, 40·0
Maternal short stature	1·0		0·03		0·02		0·11		0·005	
No	93·6	88·3, 95·5	68·3	45·5, 58·5	54·0	31·4, 44·0	17·5	6·4, 14·4	46·0	21·1, 32·7
Yes	92·5	86·1, 97·8	52·0	57·3, 77·9	37·6	42·9, 64·8	9·8	10·1, 27·2	26·6	35·2, 57·1
Maternal marital status	1·0		0·56		0·76		0·09		0·53	
With partner	92·5	88·9, 95·1	55·3	49·6, 60·9	41·6	36·1, 47·3	11·1	7·8, 15·1	31·4	26·3, 36·9
Without partner	95·2	79·3, 99·7	61·9	41·7, 79·4	38·1	20·6, 58·3	23·8	9·9, 43·7	38·1	20·6, 58·3
Number of residents in the household	0·48		0·38		0·40		0·74		0·44	
≤ 9	91·4	86·9, 94·7	58·9	52·2, 65·4	42·9	36·4, 49·7	12·3	8·3, 17·3	33·7	27·6, 40·3
≥ 10	94·0	87·7, 97·6	53·0	43·4, 62·4	37·3	28·5, 46·9	10,8	5·8, 18·2	28·9	20·8, 38·2
Place for food purchases in the community	0·74		< 0·001		< 0·001		0·09		0·006	
Yes	91·0	86·6, 94·2	75·1	69·2, 80·4	52·0	45·5, 58·4	14·7	10·5, 19·8	39·5	33·4, 46·0
No	94·1	82·6, 98·9	11·8	4·1, 24·9	8·8	2·4, 21·2	2·9	0·1, 13·2	14·7	6·0, 28·5
Residents with regular income	0·77		0·57		0·19		0·20		0·75	
Yes	91·7	86·0, 95·6	58·7	50·4, 66·7	45·9	37·7, 54·2	14·7	9·4, 21·4	33·0	25·6, 41·2
No	92·7	88·0, 96·0	55·1	47·7, 62·3	37·7	30·8, 45·0	9·4	5·7, 14·6	31·2	24·7, 38·3
Cash transfer program (*Bolsa Família*)	0·54		< 0·001		< 0·001		0·09		< 0·001	
Yes	91·1	85·0, 95·3	75·2	67·2, 82·2	54·5	45·8, 62·9	15·8	10·2, 23·1	46·5	38·0, 55·2
No	93·2	88·7, 96·3	43·5	36·6, 50·7	32·0	25·6, 38·9	8·8	5·3, 13·7	21·8	16·3, 28·1

*P* value refers to *Poisson* regression test.

In the adjusted analysis, place of residence and maternal short stature remained associated with the consumption of ultra-processed foods. The prevalence of ultra-processed food consumption was 11·6 times higher in Maturacá and 9·2 times higher in Ariabú when compared with Auaris. Ultra-processed food consumption was 31 % lower among children whose mothers had short stature than among children of mothers with adequate stature (Table 5).

**Table 5:** Crude and adjusted analysis of the association between consumption of ultra-processed foods and characteristics of indigenous children aged 6 to 59 months living in villages of the Yanomami indigenous territory, Brazil, 2018–2019 (*n* 251)

Variables	Consumption of ultra-processed foods				
	Crude PR	90 %CI	*P*-value	Adjusted PR	90 %CI
Place of residence			< 0·001		
Auaris	Ref.			Ref.	
Maturacá	5·57	2·66, 11·67		12·57	2·13, 74·13
Ariabú	6·07	2·90, 12·72		10·25	1·72, 61·19
Sex of the child			0·496	–	
Female	Ref.				
Male	1·13	0·84, 1·54			
Age of child			0·480	–	
6–23 months	Ref.				
24–59 months	1·17	0·81, 1·68			
Birth weight			0·054		
< 2500 g	Ref.			Ref.	
≥ 2500 g	2·9	1·17, 7·20		2·44	1·00, 5·89
Maternal age			0·970	–	
≤ 19 years	Ref.				
20–29 years	0·98	0·54, 1·76			
≥ 30 years	0·93	0·51, 1·71			
Maternal short stature			0·003		
No	Ref.			Ref.	
Yes	0·58	0·42, 0·78		0·69	0·51, 0·93
Maternal marital status			0·514	–	
With partner	Ref.				
Without partner	1·21	0·75, 1·97			
Number of residents in the household			0·450	–	
≤ 9	Ref.				
≥ 10	0·86	0·61, 1·20			
Place for food purchases in the community			0·020		
Yes	Ref.			Ref.	
No	0·37	0·18, 0·75		1·36	0·47, 3·95
Residents with regular income			0·755	–	
Yes	Ref.				
No	0·94	0·69, 1·28			
Cash transfer program (*Bolsa Família*)			< 0·001		
Yes	Ref.			Ref.	
No	0·47	0·34, 0·64		0·76	0·55, 1·06

*P* value refers to *Poisson* regression test.

## Discussion

The current study presents unprecedented information on the food profile of the Yanomami indigenous children. There was a high prevalence of consumption of ‘regional’ (93 %) and ‘urban’ (56 %) *in natura* or minimally processed foods. The overall prevalence of ultra-processed foods was 32 % and was associated with the place of residence and maternal stature. Moreover, a huge proportion of the children was stunted.

Few studies have been dedicated to assessing the diet consumption of Brazilian indigenous children. The First Brazilian National Survey on Indigenous People’s Health and Nutrition^(2)^, for example, evaluated only the acquisition of food and the food profile of the family/household without detailing the food consumption of the child^(27)^. Mattos *et al.* ^(13)^ reported that indigenous children from the Alto Xingu River (Brazilian mid-western region) had manioc porridge, watermelon, fruit and fish as their food base. However, Ribas *et al.* ^(16)^ revealed a diet consisting primarily of rice, manioc, sugar and meat with a high fat content among Terena indigenous children under 5 years of age. More recently, Silva *et al.* ^(28)^ observed that the dietary intake of children under 5 years of age from Karapotó (Brazilian Northeastern region) was similarly monotonous and based on rice, sugar, powdered milk and beans.

The presence of ultra-processed foods in the diet of the Brazilian indigenous children has also been described by other authors^(14,16,28,29)^. Studies conducted prior to the publication of the NOVA system did not use the term ultra-processed. Nonetheless, it is possible to infer the presence of ultra-processed foods in these previous studies because of the description of items consumed. Maciel *et al.* ^(14)^, when evaluating the indigenous people of Acre (northern region of Brazil), reported a frequency of 52·6 % and 28·6 % consumption of ultra-processed foods in children aged 6–12 months and 13–23 months, respectively. Silva *et al.* ^(28)^ reported a frequency of consumption of 33 % frankfurters, 31 % instant noodles and 27 % soft drinks among Karapotó children under 5 years of age. Ribas *et al.* ^(16)^ also observed the presence of ultra-processed foods, such as powdered soft drinks, soft drinks, candies and cookies, in the diet of Terena indigenous children. Silva *et al.* ^(29)^ noted the presence of cookies, sweets, snacks and chocolates in the diet of indigenous children from São Paulo (southeastern region of Brazil).

Previous studies suggest that the country is experiencing a transition in the eating habits of Brazilian indigenous children^(14,27)^. Traditionally, the diet of these children consisted predominantly of *in natura* foods that are available in the forest; however, there is growing access to processed foods as a result of the contact with non-indigenous population^(30)^. In a study conducted with Ecuadorian children who live in the Amazon region, high consumption of processed/ultra-processed foods was associated with a higher percentage of body fat in children^(31)^.

The prevalence of consumption of ultra-processed foods in our study was higher among children in the regions of Maturacá and Ariabú when compared with those of Auaris. Despite the geographical isolation of Yanomami indigenous communities, this can be explained by the fact that these locations have relatively easier access to urban centres, while in the Auaris region, access is more difficult and costly, since all trips are by air^(32)^. In contrast, the Auaris region has a higher frequency of cultivation and collection of wild foods than Maturacá and Ariabú^(32,33)^.

Our results also showed an association between the consumption of ultra-processed foods and maternal stature. The high prevalence of short stature in mothers (73 %) indicates the previous vulnerability to malnutrition to which these mothers are/were exposed. Previous studies carried out among Yanomami indigenous groups reported that they experience an ongoing intergenerational cycle of malnutrition^(4,34)^. Orellana *et al.* ^(4)^ revealed that the risk of having a severely short stature was 2·1 times higher in children whose mothers had short stature in two distinct Yanomami regions. Our findings suggest that Yanomami families have a high degree of socio-environmental vulnerability, which results in a permanent state of food insecurity. Considering that indigenous peoples in Brazil suffer from accumulated deficits in access to public services, such as clean water, sewage treatment and healthcare, when compared with the non-indigenous population^(1)^, it is not possible to ignore the literature that associates stunting with sanitation and subclinical illness, i.e. environmental enteric dysfunction^(35)^, diarrhoea^(36)^. Such conditions reduce the absorption of nutrients or make it impossible to absorb nutrients from the few foods they eat and, thus, affects their potential growth.

In a meta-analysis of individual data from low- and middle-income countries, mothers with short stature were more likely to give birth to babies that were small-for-gestational-age or preterm, confirming that the height-for-age deficit may begin at conception^(37)^. Intrauterine growth faltering can be observed at birth, but if socio-environmental conditions continue to be precarious, such as poor quality of food intake, the growth deficit accumulates up to 2 years of age, and it is possible to observe the maximum height-for-age deficit between 2 and 5 years of age in preschool children^(38)^.

Furthermore, our results also showed that the consumption of ultra-processed foods was 31 % lower among children of mothers with shorter stature. In this case, the short maternal stature may be a proxy of the socio-economic disadvantages, since obtaining these ultra-processed foods requires a certain level of purchasing power. Consequently, these women did not have the opportunity to ingest energy, proteins and other nutrients in sufficient amounts to achieve adequate nutritional status from any kind of food, including ultra-processed foods.

In order to meet the SDG Goal 10 ‘*Reduce inequality within and among countries*’, in governmental policies for food and nutrition security, it is critical to target disadvantaged populations^(39)^. Additionally, to minimise the chances of increasing the participation of ultra-processed foods in the diet of indigenous children, it is essential to recognise that indigenous communities need to be given priority in public policies that promote healthy eating habits through the combination of nutritional education with the improvement of access to foods and/or social support to prevent child malnutrition. Moreover, the promotion of healthy eating habits needs to target all health care programmes for the indigenous populations, as well as provide guidance to non-indigenous people that come into contact with indigenous communities. Article 11 of the International Covenant on Economic, Social and Cultural Rights^(40)^ of 1966 on the Rights of Indigenous Peoples recognises ‘*the right of everyone to an adequate standard of living…, including adequate food, housing, and continued improvement of living conditions’* and the *‘fundamental right of everyone to be free from hunger*’, including indigenous peoples. The Declaration of the Rights of Indigenous Peoples of the United Nations^(41)^, adopted by the General Assembly in 2007, recognises and stresses indigenous rights such as provisions regarding land, natural resources and subsistence activities relevant to the realisation of their right to food.

The current study has some limitations that need to be mentioned. First, the use of a pre-structured food list, which is useful for avoiding biases in information and memory bias, limits the understanding of food variability in the community, especially considering the context of seasonality, typical in the Amazon^(11,42)^. However, the use of a recall method regarding their diet may not adequately capture the exposure to ultra-processed food consumption. We acknowledge that the applied food list did not contain a specific item for infant formula, and no infant formula was recorded in the open question ‘others’. Because it is a high-cost product in Brazil and is not provided by the primary care health system, the use of infant formula is unlikely in the Yanomami indigenous population. Another limitation was the use of non-validated questionnaires for the indigenous population. However, due to the immense social diversity in Brazil, it is not possible to use any data collection instrument that is validated for wide use in different ethnic groups. At the time of the current study, no valid generalised instruments had been identified to capture data on the diet of children of indigenous groups. Future research is needed to develop and validate methods for assessing indigenous dietary intake, especially that of the children. In addition, some interviews were conducted with the support of interpreters, and the possibility of misinterpretations cannot be discarded. To minimise problems, prior to the field work, all interviewers received training to standardise data collection.

The present study has important strengths. The study was designed as a census of children living in villages in three difficult-to-access regions in the Brazilian Amazon and contributes with data on this specific and underrepresented group. Due to geographic isolation, the studied villages may spend months without receiving visits from the health teams. Therefore, increasing our knowledge of the health situation in these locations is essential. Furthermore, the way data were collected allowed the application of the NOVA system to classify foods and identify factors associated with the consumption of ultra-processed foods.

Finally, despite the high consumption of *in natura* and minimally processed foods among Yanomami indigenous children, the consumption of ultra-processed foods was also high and was associated with the area of residence as a proxy for access to food from contact with people of the non-indigenous population and with mothers of adequate stature. The current study also emphasises the need for culturally acceptable programmes and interventions that promote complementary food education actions for families with chronic nutritional deficits, especially in areas close to indigenous territories, and consumer protection policies that guarantee consumers information on the harmful effects of the consumption of ultra-processed foods^(43,44)^.

## Supplementary material

For supplementary material accompanying this paper visit https://doi.org/10.1017/S1368980022001306sup001. click here to view supplementary material

## References

[1] Coimbra CEA , Santos RV , Welch JR et al. (2013) The first national survey of indigenous people’s health and nutrition in Brazil: rationale, methodology, and overview of results. BMC Public Health 13, 52. 23331985 10.1186/1471-2458-13-52 PMC3626720

[2] Horta BL , Santos RV , Welch JR et al. (2013) Nutritional status of indigenous children: findings from the first national survey of indigenous people’s health and nutrition in Brazil. Int J Equity Health 12, 23. 23552397 10.1186/1475-9276-12-23 PMC3637628

[3] Pantoja LN , Orellana JDY , Leite MS et al. (2014) The coverage of the System for Nutrition Surveillance of Indigenous Peoples (SISVAN-I) and the prevalence of nutritional disorders in Yanomami children aged under 60 months, Amazonia, Brazil. Rev Bras Saude Mater Infant 14, 53–63.

[4] Orellana JDY , Marrero L , Alves CLM et al. (2019) Association of severe stunting in indigenous Yanomami children with maternal short stature: clues about the intergerational transmission. Cienc Saude Colet 24, 1875–1883. 10.1590/1413-81232018245.17062017 31166520

[5] Alves DF (2017) Povos indígenas, juventude e direitos violados na Amazônia Brasileira (Indigenous peoples, youth and violated rights in the Brazilian Amazon). Juv Indíg 22640, 142–153.

[6] Albert B (1992) A fumaça do metal: história e representações do contato entre os yanomami (The smoke of metal: history and representations of contact among the Yanomami). Anu Antropol 89, 151–190.

[7] Survival International (2019) The Yanomami. https://www.survivalinternational.org/tribes/yanomami (accessed February 2022).

[8] Pontes BMS (2019) Movimento de resistência socioterritorial nas terras indígenas yanomami (Social-territorial resistance movement on Yanomami indigenous lands). Rev Mov Soc Din Espaciais 8, 82–104.

[9] Ramos ARA , Abrahão BA & Rodrigues FS (2020) Absence of state power in artesanal mining developed inside yanomami indigenous land – Brazilian Amazon. Braz J Dev 6, 15753–15771.

[10] Leite MS (2007) Transformação e Persistência: Antropologia da Alimentação e Nutrição em Uma Sociedade Indígena Amazônica (Transformation and Persistence: Anthropology of Food and Nutrition in an Amazonian Indigenous Society). Rio de Janeiro: Fundação Oswaldo Cruz.

[11] Gatica-Domínguez G , Mesenburg MA , Barros AJD et al. (2020) Ethnic inequalities in child stunting and feeding practices: results from surveys in thirteen countries from Latin America. Int J Equity Health 19, 53. 32272935 10.1186/s12939-020-01165-9 PMC7147069

[12] Neitzel AL , Smalls BL , Walker RJ et al. (2019) Examination of dietary habits among the indigenous Kuna Indians of Panama. Nutr J 18, 1–8. 31370836 10.1186/s12937-019-0469-8 PMC6670206

[13] Mattos A , Morais MB , Rodrigues DA et al. (1999) Nutritional status and dietary habits of Indian children from Alto Xingu (Central Brazil) according to age. J Am Coll Nutr 18, 88–94. 10067664 10.1080/07315724.1999.10718832

[14] Maciel VBS , Coca KP , Castro LS et al. (2021) Food diversity among indigenous children from two municipalities of the Brazilian Western Amazon. Cienc Saude Colet 26, 2921–2928. 10.1590/1413-81232021267.14232019 34231704

[15] Serafim MG (1997) Hábitos alimentares e nível de hemoglobina em crianças indígenas Guarani, menores de 5 anos dos Estados de São Paulo e do Rio de Janeiro (Dietary habits and hemoglobin level in Guarani indigenous children under 5 years of age in the states of São Paulo and Rio de Janeiro). Master’s Dissertation, Universidade Federal de São Paulo.

[16] Ribas DLB , Sganzerla A , Zorzatto JR et al. (2001) Child health and nutrition in a Teréna indigenous community, Mato Grosso do Sul, Brazil. Cad Saude Publica 17, 323–331. 11283763 10.1590/s0102-311x2001000200007

[17] Bortolini GA , Gubert MB & Santos LMP (2012) Food consumption Brazilian children by 6 to 59 months of age. Cad Saude Publica 29, 1759–1771. 10.1590/s0102-311x2012000900014 23033190

[18] Mello CS , Barros KV & Morais MB (2016) Brazilian infant and preschool children feeding: literature review. J Pediatr 92, 451–463. 10.1016/j.jped.2016.02.013 27320201

[19] United Nations (2021) Sustainable Development. https://sdgs.un.org/goals (accessed March 2022).

[20] Magalhães ED & Cavalcanti L (1998) Morbi-Mortalidade Yanomami – 1991 a 1997. Boa Vista, RR: UFRR/FIOCRUZ.

[21] Monteiro CA , Cannon G , Levy R et al. (2019) Ultra-processed foods: what they are and how to identify them. Public Health Nutr 22, 936–941. 10.1017/S1368980018003762 PMC10260459 30744710

[22] WHO Expert Committee on Physical Status (1995) The Use and Interpretation of Anthropometry. Geneva: WHO. 8594834

[23] Ministério da Saúde (2004) Food and Nutritional Surveillance – Sisvan. Basic Guidance for Data Collection, Processing and Analysis and Information on Health Services. Brasília: Ministério da Saúde.

[24] World Health Organization (2006) WHO Child Growth Standards: Length/Height-for-Age, Weight-for-Age, Weight-for-Length, Weight-for-Height and Body Mass Index-for-Age. Methods and Development. Geneva: WHO.

[25] World Health Organization (1995) Maternal anthropometry and pregnancy outcomes: a WHO collaborative study. Bull World Health Organ 73, 32–37. 8529277 PMC2486648

[26] Lane MM , Davis AJ , Beattie S et al. (2020) Ultraprocessed food and chronic non-communicable diseases: a systematic review and meta-analysis of 43 observational studies. Obes Rev 22, 1–19. 10.1111/obr.13146 33167080

[27] Welch JR , Ferreira AA , Souza MC et al. (2021) Food profiles of indigenous households in Brazil: results of the first national survey of indigenous peoples’ health and nutrition. Ecol Food Nutr 60, 4–24. 33573410 10.1080/03670244.2020.1781105

[28] Silva DAV (2014) Consumo alimentar e estado nutricional de criança da etnia karapató em Alagoas (Food consumption and nutritional status of children of the Karapató ethnic group in Alagoas). Master's Dissertation, Universidade Federal de Alagoas.

[29] Silva LM (2013) O aleitamento materno e a alimentação infantil entre os indígenas da região oeste do estado de São Paulo: um movimento entre a tradição e interculturalidade (Breastfeeding and infant feeding among indigenous people in the western region of the state of São Paulo: a movement between tradition and interculturalism). Doctorate Thesis, Universidade de São Paulo.

[30] Eloy L (2009) Diversidade alimentar e urbanização: o papel das migrações circulares indígenas no Noroeste Amazônico (Food diversity and urbanization: the role of indigenous circular migrations in the Northwest Amazon). Anthropol Food S6, e39.

[31] Urlacher SS , Snodgrass JJ , Dugas LR et al. (2021) Childhood daily energy expenditure does not decrease with market integration and is not related to adiposity in Amazonia. J Nutr 151, 695–704. 33454748 10.1093/jn/nxaa361

[32] Basta PC & Orellana JDY (2020) Pesquisa Sobre os Determinantes Sociais da Desnutrição de Crianças Indígenas de Até 5 Anos de idade Em Oito Aldeias Inseridas no Distrito Sanitário Especial Indígena (DSEI) Yanomami (Research on the Social Determinants of Malnutrition in Indigenous Children Up to 5 Years of Age in Eight Villages Within the Special Indigenous Health District (DSEI) Yanomami). Rio de Janeiro: Fundação Oswaldo Cruz, United Nations Children’s Fund, UNICEF.

[33] Lizot J (1980) La agricultura yanõmami (Yanomami agriculture). Antropológica 53, 3–93.

[34] Orellana JDY , Domínguez GG , Vaz JS et al. (2021) Intergenerational association of short maternal stature with stunting in yanomami indigenous children from the Brazilian Amazon. Int J Environ Res Public Health 18, 1–14. 10.3390/ijerph18179130 PMC8430951 34501720

[35] Budge S , Parker AH , Hutchings PT et al. (2019) Environmental enteric dysfunction and child stunting. Nutr Rev 77, 240–253. 30753710 10.1093/nutrit/nuy068 PMC6394759

[36] Escobar AL , Coimbra CE Jr , Welch JR et al. (2015) Diarrhea and health inequity among Indigenous children in Brazil: results from the first national survey of indigenous people’s health and nutrition. BMC Public Health 15, 1–11. 25880758 10.1186/s12889-015-1534-7 PMC4349470

[37] Kozuki N , Katz J , Lee ACC et al. (2015) Child health epidemiology reference group small-for-gestational-age/preterm birth working group, short maternal stature increases risk of small-for-gestational-age and preterm births in low- and middle-income countries: individual participant data meta-analysis and population attributable fraction. J Nutr 145, 2542–2550, 26423738 10.3945/jn.115.216374 PMC6457093

[38] Victora CG , Christian P , Vidaletti LP et al. (2021) Revisiting maternal and child undernutrition in low-income and middle-income countries: variable progress towards an unfinished agenda. Lancet 397, 1388–1399. 33691094 10.1016/S0140-6736(21)00394-9 PMC7613170

[39] Mayén AL , Mestral C , Zamora G et al. (2016) Interventions promoting healthy eating as a tool for reducing social inequalities in diet in low- and middle-income countries: a systematic review. Int J Equity Health 15, 1–10. 28007023 10.1186/s12939-016-0489-3 PMC5180409

[40] United Nations (1967) International Covenant on Economic, Social and Cultural Rights. New York: ONU.

[41] UN General Assembly (2007) United Nations Declaration on the Rights of Indigenous Peoples: Resolution/Adopted by the General Assembly. https://www.refworld.org/docid/471355a82.html (accessed March 2022).

[42] Leite MS , Santos RV & Coimbra CEA Jr (2007) Sazonalidade e estado nutricional de populações indígenas: o caso Wari’, Rondônia, Brasil (Seasonality and nutritional status of indigenous peoples: the case of Wari’ in Rondônia State, Brazil). Cad Saude Publica 23, 2631–2642. 17952256 10.1590/s0102-311x2007001100011

[43] Costa CS , Del-Ponte B , Assunção MCF et al. (2018) Consumption of ultra-processed foods and body fat during childhood and adolescence: a systematic review. Public Health Nutr 21, 148–159. 28676132 10.1017/S1368980017001331 PMC10260745

[44] Costa CS , Rauber F , Leffa OS et al. (2019) Ultra-processed food consumption and its effects on anthropometric and glucose profile: a longitudinal study during childhood. Nutr Metab Cardiovasc Dis 29, 177–184. 30660687 10.1016/j.numecd.2018.11.003

