# Supplementary material for: Food profile of Yanomami indigenous children aged 6 to 59 months from the Brazilian Amazon, according to the degree of food processing: a cross-sectional study
Source: Public Health Nutr. 2022 May 27;26(1):208–18. doi: 10.1017/S1368980022001306 (PMC11077455; doi:10.1017/S1368980022001306)
Supplement: Supplementary file 1 [file S1368980022001306sup001.docx]

SUPPLEMENTARY MATERIAL

**Moraes *et al.***

**Food profile of Yanomami indigenous children aged 6 to 59 months from the Brazilian Amazon, according to the degree of food processing: a cross-sectional study**

*****STATISTICAL ANALISYS***** STATA script

***Table 2. Descriptive Analysis: Socioeconomic, demographic, maternal and individual characteristics***

tab PLACE_RESIDENCE // place of residence (auaris, maturaca, ariabu)

tab SEX_CHILD// Sex of the child

tab CHILD_AGE // Age of child

tab BIRTHWEIGHT // Birth weight (<2,500g; >=2,500g)

tab stunded_child// Stunted (y/n)

tab bmi_for_age_child // Body mass index-for-age

tab MATERNAL_AGE // Maternal age

tab SHORTSTATURE_MATERNAL // Maternal short stature

tab MARITAL_STATUS // Maternal marital status

tab N_RESIDENTS // Number of residents in the household

tab PURCHASEFOODS // Place for food purchases in the community

tab REGULAR_INCOME // Residents with paid work

tab CASHTRANSFER // Cash transfer program (Bolsa Família)

***Table 3. Descriptive Analysis: Frequency of food consumption according to the degree of food processing - Total and for age groupe (6-23 months==1 and 24-59 months==2)***

//In natura or minimally processed (regional): Fruits (Pineapple, açaí, banana, cocoa, coconut, cupuaçu, guava, ingá, orange, watermelon, passion and tucumã); Corn, roots or tubersb (Corn, manioc, manioc flour, and yam/sweet potato); Peach palm or palm heart; Fish or crab; Biju; Wild meat (Tapir, snake, agouti, monkey, curassow, namboo, paca, wild pig, toad, and deer); Chibé (manioc flour soup); Sugar cane; Mushroom; Porridges (banana, cassava flour, corn); Others (Breast milk, vegetables and legumes, ant or larvae, honey, natural fruit juice, earthworm, pepper, vegetable/fish broth and chestnuts)//

tab INNATURA_REGIONAL

tab INNATURA_REGIONAL if CHILD_AGE==1

tab INNATURA_REGIONAL if CHILD_AGE==2

tab FRUITS

tab FRUITS if CHILD_AGE==1

tab FRUITS if CHILD_AGE==2

tab ROOTS_REGIONAL

tab ROOTS_REGIONALif CHILD_AGE==1

tab ROOTS_REGIONALif CHILD_AGE==2

tab PUPUNPALM

tab PUPUNPALM if CHILD_AGE==1

tab PUPUNPALM if CHILD_AGE==2

tab FISH_CRAB

tab FISH_CRAB if CHILD_AGE==1

tab FISH_CRAB if CHILD_AGE==2

tab BIJU

tab BIJU if CHILD_AGE==1

tab BIJU if CHILD_AGE==2

tab WILD_MEAT

tab WILD_MEAT if CHILD_AGE==1

tab WILD_MEAT if CHILD_AGE==2

tab CHIBE

tab CHIBE if CHILD_AGE==1

tab CHIBE if CHILD_AGE==2

tab SUGARCANE

tab SUGARCANE if CHILD_AGE==1

tab SUGARCANE if CHILD_AGE==2

tab MUSHROOM

tab MUSHROOM if CHILD_AGE==1

tab MUSHROOM if CHILD_AGE==2

tab PORRIDGES

tab PORRIDGES if CHILD_AGE==1

tab PORRIDGES if CHILD_AGE==2

//In natura or minimally processed (urban): Beans; Rice or pasta; Chicken; Coffee or coffee with milk; Cow’s milk powder; Others (Beef or egg soup, oatmeal)//

tab INNATURA_URBAN

tab INNATURA_URBAN if CHILD_AGE==1

tab INNATURA_URBAN if CHILD_AGE==2

tab BLACK_BEANS

tab BLACK_BEANS if CHILD_AGE==1

tab BLACK_BEANS if CHILD_AGE==2

tab RICE_PASTA

tab RICE_PASTA if CHILD_AGE==1

tab RICE_PASTA if CHILD_AGE==2

tab CHICKEN

tab CHICKENif CHILD_AGE==1

tab CHICKENif CHILD_AGE==2

tab COFFEE

tab COFFEE if CHILD_AGE==1

tab COFFEE if CHILD_AGE==2

tab MILK_POWDER

tab MILK_POWDER if CHILD_AGE==1

tab MILK_POWDER if CHILD_AGE==2

//Processed culinary ingredients: Sugar; Table salt; Vegetable oil//

tab CULINARY_INGR

tab CULINARY_INGR if CHILD_AGE==1

tab CULINARY_INGR if CHILD_AGE==2

tab SUGAR

tab SUGAR if CHILD_AGE==1

tab SUGAR if CHILD_AGE==2

tab SALT

tab SALT if CHILD_AGE==1

tab SALT if CHILD_AGE==2

tab VEG_OIL

tab VEG_OIL if CHILD_AGE==1

tab VEG_OIL if CHILD_AGE==2

//Processed foods: Canned foods, processed meat; Bread//

tab PROCESS

tab PROCESS if CHILD_AGE==1

tab PROCESS if CHILD_AGE==2

tab CANNED_MEAT

tab CANNED_MEAT if CHILD_AGE==1

tab CANNED_MEAT if CHILD_AGE==2

tab BREAD

tab BREAD if CHILD_AGE==1

tab BREAD if CHILD_AGE==2

//Ultra-processed foods: Cakes or cookies; Chocolate or chocolate powder; Artificial juice or soft drink; Noodles//

tab ULTRAPROCESS

tab ULTRAPROCESS if CHILD_AGE==1

tab ULTRAPROCESS if CHILD_AGE==2

tab CAKES_COOKIES

tab CAKES_COOKIES if CHILD_AGE==1

tab CAKES_COOKIES if CHILD_AGE==2

tab CHOCOLATE

tab CHOCOLATE if CHILD_AGE==1

tab CHOCOLATE if CHILD_AGE==2

tab JUICE_ART_SOFTD

tab JUICE_ART_SOFTD if CHILD_AGE==1

tab JUICE_ART_SOFTD if CHILD_AGE==2

tab NOODLES

tab NOODLES if CHILD_AGE==1

tab NOODLES if CHILD_AGE==2

***Table 4. Frequency of food consumption according to the degree of food processing according to selected Socioeconomic, demographic, maternal and individual characteristics

//Current place of residence; Sex of the child; Age of child; Birth weight; Maternal age; Maternal short stature; Maternal marital status; Number of residents in the household; Place for food purchases in the community; Residents with paid work; Cash transfer program (Bolsa Família)//

*Pearson’s chi-square or Fisher’s: Significance = P-value<0,10; 90% confidence intervals*

//In natura or minimally processed (Urban)//

tab PLACE_RESIDENCE INNATURA_REGIONAL, row chi2 exact

ci INNATURA_REGIONAL if PLACE_RESIDENCE==1, binomial level (90)

ci INNATURA_REGIONAL if PLACE_RESIDENCE==2, binomial level (90)

ci INNATURA_REGIONAL if PLACE_RESIDENCE==3, binomial level (90)

tab N_RESIDENTS INNATURA_REGIONAL, row chi2 exact

ci INNATURA_REGIONAL if N_RESIDENTS==1, binomial level (90)

ci INNATURA_REGIONAL if N_RESIDENTS==2, binomial level (90)

tab REGULAR_INCOME INNATURA_REGIONAL, row chi2 exact

ci INNATURA_REGIONAL if REGULAR_INCOME==1, binomial level (90)

ci INNATURA_REGIONAL if REGULAR_INCOME==2, binomial level (90)

tab CASHTRANSFER INNATURA_REGIONAL, row chi2 exact

ci INNATURA_REGIONAL if CASHTRANSFER==1, binomial level (90)

ci INNATURA_REGIONAL if CASHTRANSFER==2, binomial level (90)

tab PURCHASEFOODS INNATURA_REGIONAL, row chi2 exact

ci INNATURA_REGIONAL if PURCHASEFOODS==1, binomial level (90)

ci INNATURA_REGIONAL if PURCHASEFOODS==2, binomial level (90)

tab MARITAL_STATUS INNATURA_REGIONAL, row chi2 exact

ci INNATURA_REGIONAL if MARITAL_STATUS==1, binomial level (90)

ci INNATURA_REGIONAL if MARITAL_STATUS==2, binomial level (90)

tab MATERNAL_AGE INNATURA_REGIONAL, row chi2 exact

ci INNATURA_REGIONAL if MATERNAL_AGE==1, binomial level (90)

ci INNATURA_REGIONAL if MATERNAL_AGE==2, binomial level (90)

ci INNATURA_REGIONAL if MATERNAL_AGE==3, binomial level (90)

tab SHORTSTATURE_MATERNAL INNATURA_REGIONAL, row chi2 exact

ci INNATURA_REGIONAL if SHORTSTATURE_MATERNAL==1, binomial level (90)

ci INNATURA_REGIONAL if SHORTSTATURE_MATERNAL==2, binomial level (90)

tab CHILD_AGE INNATURA_REGIONAL, row chi2 exact

ci INNATURA_REGIONAL if CHILD_AGE==1, binomial level (90)

ci INNATURA_REGIONAL if CHILD_AGE==2, binomial level (90)

tab SEX_CHILDINNATURA_REGIONAL, row chi2 exact

ci INNATURA_REGIONAL if SEXO_OK==1, binomial level (90)

ci INNATURA_REGIONAL if SEXO_OK==2, binomial level (90)

tab BIRTHWEIGHT INNATURA_REGIONAL, row chi2 exact

ci INNATURA_REGIONAL if BIRTHWEIGHT==1, binomial level (90)

ci INNATURA_REGIONAL if BIRTHWEIGHT==2, binomial level (90)

//In natura or minimally processed (Urban)//

tab PLACE_RESIDENCE INNATURA_URBAN, row chi2 exact

ci INNATURA_URBAN if PLACE_RESIDENCE==1, binomial level (90)

ci INNATURA_URBAN if PLACE_RESIDENCE==2, binomial level (90)

ci INNATURA_URBAN if PLACE_RESIDENCE==3, binomial level (90)

tab N_RESIDENTS INNATURA_URBAN, row chi2 exact

ci INNATURA_URBAN if N_RESIDENTS==1, binomial level (90)

ci INNATURA_URBAN if N_RESIDENTS==2, binomial level (90)

tab REGULAR_INCOME INNATURA_URBAN, row chi2 exact

ci INNATURA_URBAN if REGULAR_INCOME==1, binomial level (90)

ci INNATURA_URBAN if REGULAR_INCOME==2, binomial level (90)

tab CASHTRANSFER INNATURA_URBAN, row chi2 exact

ci INNATURA_URBAN if CASHTRANSFER==1, binomial level (90)

ci INNATURA_URBAN if CASHTRANSFER==2, binomial level (90)

tab PURCHASEFOODS INNATURA_URBAN, row chi2 exact

ci INNATURA_URBAN if PURCHASEFOODS==1, binomial level (90)

ci INNATURA_URBAN if PURCHASEFOODS==2, binomial level (90)

tab MARITAL_STATUS INNATURA_URBAN, row chi2 exact

ci INNATURA_URBAN if MARITAL_STATUS==1, binomial level (90)

ci INNATURA_URBAN if MARITAL_STATUS==2, binomial level (90)

tab MATERNAL_AGE INNATURA_URBAN, row chi2 exact

ci INNATURA_URBAN if MATERNAL_AGE==1, binomial level (90)

ci INNATURA_URBAN if MATERNAL_AGE==2, binomial level (90)

ci INNATURA_URBAN if MATERNAL_AGE==3, binomial level (90)

tab SHORTSTATURE_MATERNAL INNATURA_URBAN, row chi2 exact

ci INNATURA_URBAN if SHORTSTATURE_MATERNAL==1, binomial level (90)

ci INNATURA_URBAN if SHORTSTATURE_MATERNAL==2, binomial level (90)

tab CHILD_AGE INNATURA_URBAN, row chi2 exact

ci INNATURA_URBAN if CHILD_AGE==1, binomial level (90)

ci INNATURA_URBAN if CHILD_AGE==2, binomial level (90)

tab SEX_CHILDINNATURA_URBAN, row chi2 exact

ci INNATURA_URBAN if SEXO_OK==1, binomial level (90)

ci INNATURA_URBAN if SEXO_OK==2, binomial level (90)

tab BIRTHWEIGHT INNATURA_URBAN, row chi2 exact

ci INNATURA_URBAN if BIRTHWEIGHT==1, binomial level (90)

ci INNATURA_URBAN if BIRTHWEIGHT==2, binomial level (90)

//Processed culinary ingredients//

tab PLACE_RESIDENCE CULINARY_INGR, row chi2 exact

ci CULINARY_INGR if PLACE_RESIDENCE==1, binomial level (90)

ci CULINARY_INGR if PLACE_RESIDENCE==2, binomial level (90)

ci CULINARY_INGR if PLACE_RESIDENCE==3, binomial level (90)

tab N_RESIDENTS CULINARY_INGR, row chi2 exact

ci CULINARY_INGR if N_RESIDENTS==1, binomial level (90)

ci CULINARY_INGR if N_RESIDENTS==2, binomial level (90)

tab REGULAR_INCOME CULINARY_INGR, row chi2 exact

ci CULINARY_INGR if REGULAR_INCOME==1, binomial level (90)

ci CULINARY_INGR if REGULAR_INCOME==2, binomial level (90)

tab CASHTRANSFER CULINARY_INGR, row chi2 exact

ci CULINARY_INGR if CASHTRANSFER==1, binomial level (90)

ci CULINARY_INGR if CASHTRANSFER==2, binomial level (90)

tab PURCHASEFOODS CULINARY_INGR, row chi2 exact

ci CULINARY_INGR if PURCHASEFOODS==1, binomial level (90)

ci CULINARY_INGR if PURCHASEFOODS==2, binomial level (90)

tab MARITAL_STATUS INNATURA_URBAN, row chi2 exact

ci INNATURA_URBAN if MARITAL_STATUS==1, binomial level (90)

ci INNATURA_URBAN if MARITAL_STATUS==2, binomial level (90)

tab MATERNAL_AGE CULINARY_INGR, row chi2 exact

ci CULINARY_INGR if MATERNAL_AGE==1, binomial level (90)

ci CULINARY_INGR if MATERNAL_AGE==2, binomial level (90)

ci CULINARY_INGR if MATERNAL_AGE==3, binomial level (90)

tab SHORTSTATURE_MATERNAL CULINARY_INGR, row chi2 exact

ci CULINARY_INGR if SHORTSTATURE_MATERNAL==1, binomial level (90)

ci CULINARY_INGR if SHORTSTATURE_MATERNAL==2, binomial level (90)

tab CHILD_AGE CULINARY_INGR, row chi2 exact

ci CULINARY_INGR if CHILD_AGE==1, binomial level (90)

ci CULINARY_INGR if CHILD_AGE==2, binomial level (90)

tab SEX_CHILDCULINARY_INGR, row chi2 exact

ci CULINARY_INGR if SEXO_OK==1, binomial level (90)

ci CULINARY_INGR if SEXO_OK==2, binomial level (90)

tab BIRTHWEIGHT CULINARY_INGR, row chi2 exact

ci CULINARY_INGR if BIRTHWEIGHT==1, binomial level (90)

ci CULINARY_INGR if BIRTHWEIGHT==2, binomial level (90)

//Processed foods//

tab PLACE_RESIDENCE PROCESS, row chi2 exact

ci PROCESS if PLACE_RESIDENCE==1, binomial level (90)

ci PROCESS if PLACE_RESIDENCE==2, binomial level (90)

ci PROCESS if PLACE_RESIDENCE==3, binomial level (90)

tab N_RESIDENTS PROCESS, row chi2 exact

ci PROCESS if N_RESIDENTS==1, binomial level (90)

ci PROCESS if N_RESIDENTS==2, binomial level (90)

tab REGULAR_INCOME PROCESS, row chi2 exact

ci PROCESS if REGULAR_INCOME==1, binomial level (90)

ci PROCESS if REGULAR_INCOME==2, binomial level (90)

tab CASHTRANSFER PROCESS, row chi2 exact

ci PROCESS if CASHTRANSFER==1, binomial level (90)

ci PROCESS if CASHTRANSFER==2, binomial level (90)

tab PURCHASEFOODS PROCESS, row chi2 exact

ci PROCESS if PURCHASEFOODS==1, binomial level (90)

ci PROCESS if PURCHASEFOODS==2, binomial level (90)

tab MARITAL_STATUS PROCESS, row chi2 exact

ci PROCESS if MARITAL_STATUS==1, binomial level (90)

ci PROCESS if MARITAL_STATUS==2, binomial level (90)

tab MATERNAL_AGE PROCESS, row chi2 exact

ci PROCESS if MATERNAL_AGE==1, binomial level (90)

ci PROCESS if MATERNAL_AGE==2, binomial level (90)

ci PROCESS if MATERNAL_AGE==3, binomial level (90)

tab SHORTSTATURE_MATERNAL PROCESS, row chi2 exact

ci PROCESS if SHORTSTATURE_MATERNAL==1, binomial level (90)

ci PROCESS if SHORTSTATURE_MATERNAL==2, binomial level (90)

tab CHILD_AGE PROCESS, row chi2 exact

ci PROCESS if CHILD_AGE==1, binomial level (90)

ci PROCESS if CHILD_AGE==2, binomial level (90)

tab SEX_CHILDPROCESS, row chi2 exact

ci PROCESS if SEXO_OK==1, binomial level (90)

ci PROCESS if SEXO_OK==2, binomial level (90)

tab BIRTHWEIGHT PROCESS, row chi2 exact

ci PROCESS if BIRTHWEIGHT==1, binomial level (90)

ci PROCESS if BIRTHWEIGHT==2, binomial level (90)

//Ultra-processed foods//

tab PLACE_RESIDENCE ULTRAPROCESS, row chi2 exact

ci ULTRAPROCESS if PLACE_RESIDENCE==1, binomial level (90)

ci ULTRAPROCESS if PLACE_RESIDENCE==2, binomial level (90)

ci ULTRAPROCESS if PLACE_RESIDENCE==3, binomial level (90)

tab N_RESIDENTS ULTRAPROCESS, row chi2 exact

ci ULTRAPROCESS if N_RESIDENTS==1, binomial level (90)

ci ULTRAPROCESS if N_RESIDENTS==2, binomial level (90)

tab REGULAR_INCOME ULTRAPROCESS, row chi2 exact

ci ULTRAPROCESS if REGULAR_INCOME==1, binomial level (90)

ci ULTRAPROCESS if REGULAR_INCOME==2, binomial level (90)

tab CASHTRANSFER ULTRAPROCESS, row chi2 exact

ci ULTRAPROCESS if CASHTRANSFER==1, binomial level (90)

ci ULTRAPROCESS if CASHTRANSFER==2, binomial level (90)

tab PURCHASEFOODS ULTRAPROCESS, row chi2 exact

ci ULTRAPROCESS if PURCHASEFOODS==1, binomial level (90)

ci ULTRAPROCESS if PURCHASEFOODS==2, binomial level (90)

tab MARITAL_STATUS ULTRAPROCESS, row chi2 exact

ci ULTRAPROCESS if MARITAL_STATUS==1, binomial level (90)

ci ULTRAPROCESS if MARITAL_STATUS==2, binomial level (90)

tab MATERNAL_AGE ULTRAPROCESS, row chi2 exact

ci ULTRAPROCESS if MATERNAL_AGE==1, binomial level (90)

ci ULTRAPROCESS if MATERNAL_AGE==2, binomial level (90)

ci ULTRAPROCESS if MATERNAL_AGE==3, binomial level (90)

tab SHORTSTATURE_MATERNAL ULTRAPROCESS, row chi2 exact

ci ULTRAPROCESS if SHORTSTATURE_MATERNAL==1, binomial level (90)

ci ULTRAPROCESS if SHORTSTATURE_MATERNAL==2, binomial level (90)

tab CHILD_AGE ULTRAPROCESS, row chi2 exact

ci ULTRAPROCESS if CHILD_AGE==1, binomial level (90)

ci ULTRAPROCESS if CHILD_AGE==2, binomial level (90)

tab SEX_CHILDULTRAPROCESS, row chi2 exact

ci ULTRAPROCESS if SEXO_OK==1, binomial level (90)

ci ULTRAPROCESS if SEXO_OK==2, binomial level (90)

tab BIRTHWEIGHT ULTRAPROCESS, row chi2 exact

ci ULTRAPROCESS if BIRTHWEIGHT==1, binomial level (90)

ci ULTRAPROCESS if BIRTHWEIGHT==2, binomial level (90)

***Table 5. Crude and adjusted analysis of the association between consumption of ultra-processed foods and socioeconomic, demographic, maternal and individual characteristics***

*Poisson regression analysis with robust variances, estimating the crude and adjusted prevalence ratios with 90% CI*

//Crude analisys//

poisson ULTRAPROCESS i.PLACE_RESIDENCE, robust irr level (90)

poisson ULTRAPROCESS N_RESIDENTS, robust irr level (90)

poisson ULTRAPROCESS REGULAR_INCOME, robust irr level (90)

poisson ULTRAPROCESS CASHTRANSFER, robust irr level (90)

poisson ULTRAPROCESS PURCHASEFOODS, robust irr level (90)

poisson ULTRAPROCESS MARITAL_STATUS, robust irr level (90)

poisson ULTRAPROCESS i.MATERNAL_AGE, robust irr level (90)

poisson ULTRAPROCESS SHORTSTATURE_MATERNAL, robust irr level (90)

poisson ULTRAPROCESS CHILD_AGE, robust irr level (90)

poisson ULTRAPROCESS SEXO_OK, robust irr level (90)

poisson ULTRAPROCESS BIRTHWEIGHT, robust irr level (90)

//Adjusted analisys//

*Note: Variables with p-values <0.20 in the bivariate analysis were included in the multivariate analysis*

poisson ULTRAPROCESS i.PLACE_RESIDENCE CASHTRANSFER PURCHASEFOODS SHORTSTATURE_MATERNAL BIRTHWEIGHT, robust irr level (90)
